# Supplementary figures and images for: Comprehensive analysis of COMMD10 as a novel prognostic biomarker for gastric cancer
Source: PeerJ. 2023 Mar 9;11:e14645. doi: 10.7717/peerj.14645 (PMC10008319; doi:10.7717/peerj.14645)

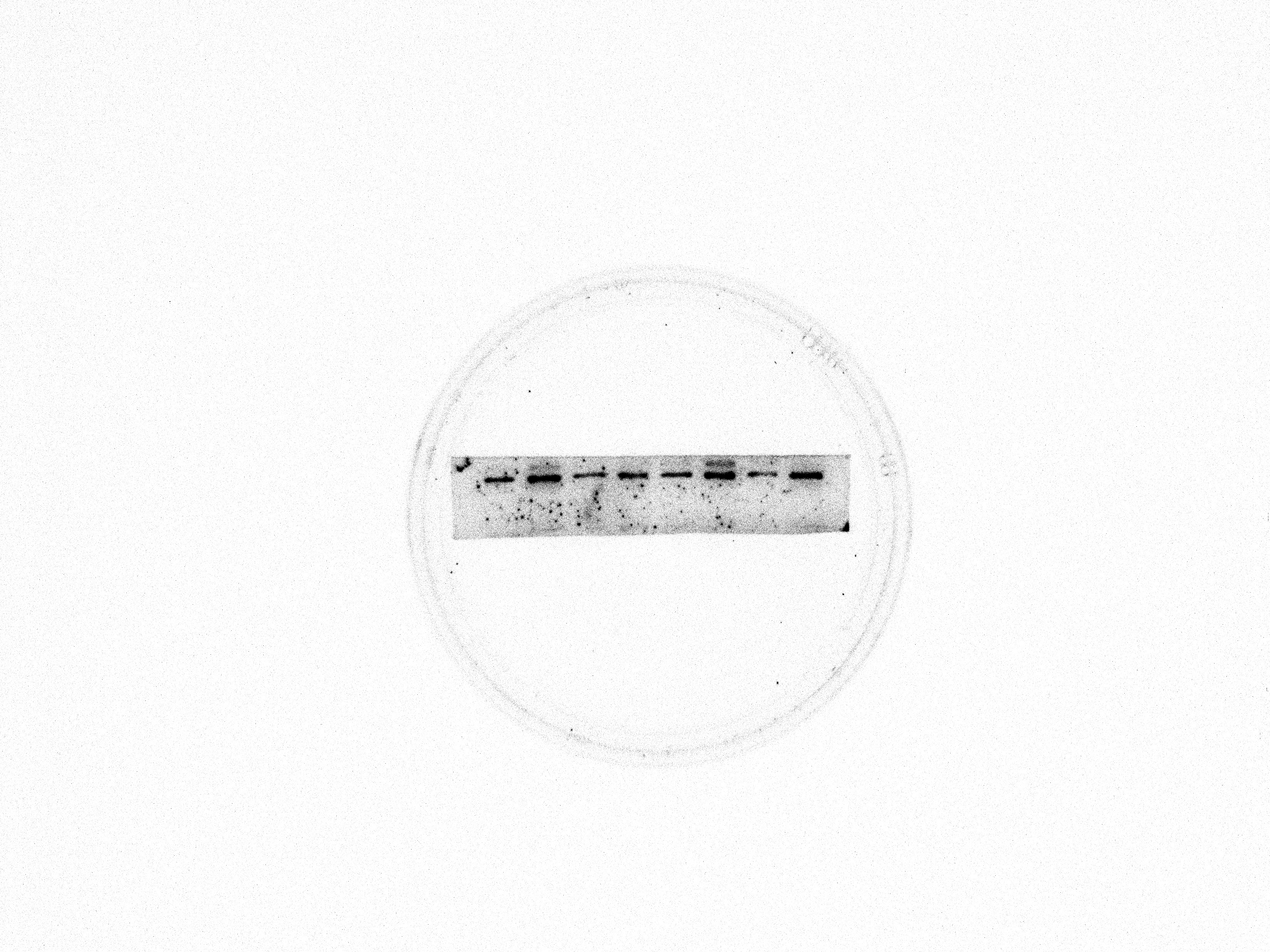

Supplement: Supplemental Information 1 [file peerj-11-14645-s001.zip › additional files/Figure 1E COMM10 (1).jpg]

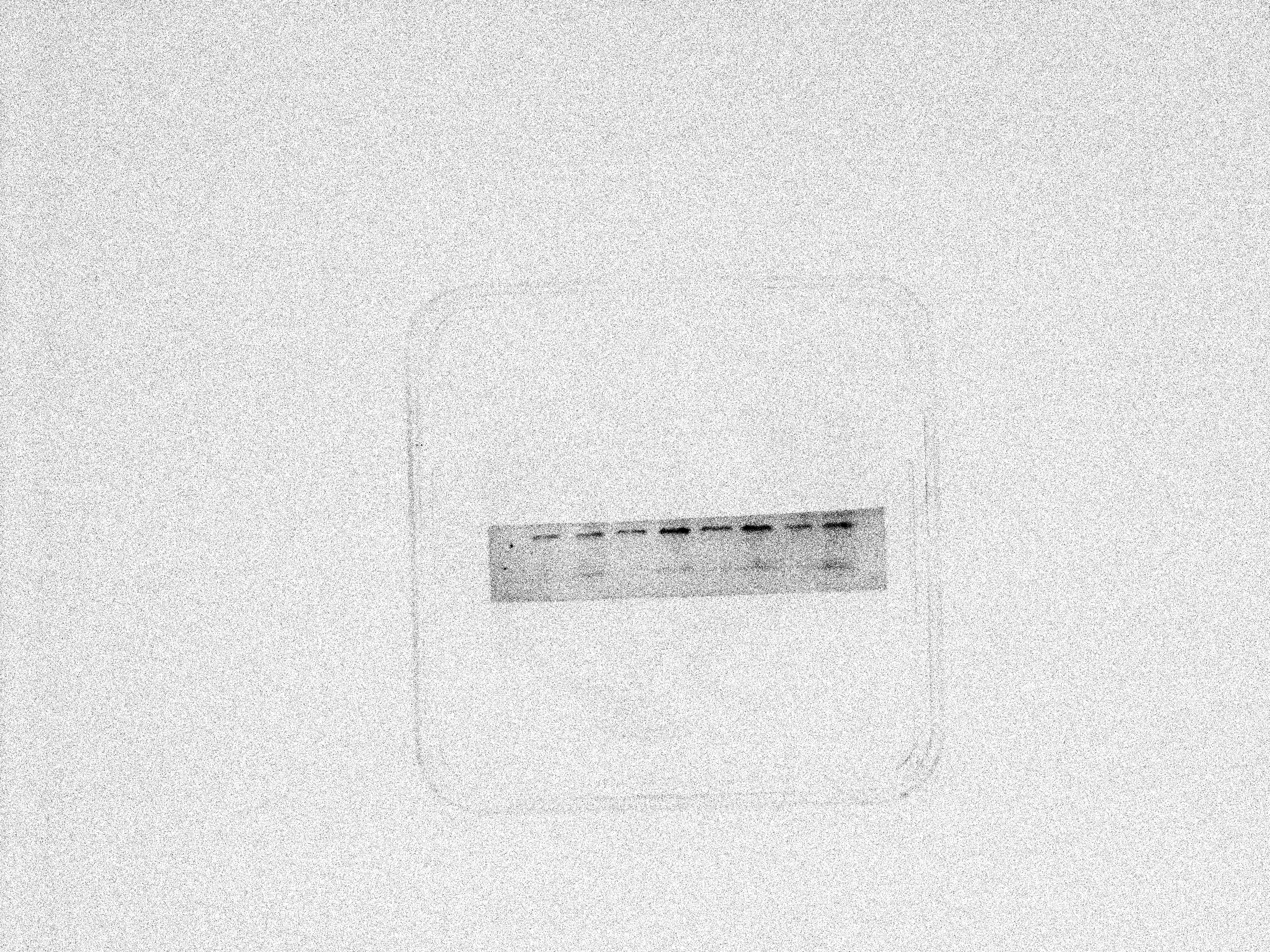

Supplement: Supplemental Information 1 [file peerj-11-14645-s001.zip › additional files/Figure 1E COMM10 (2).jpg]

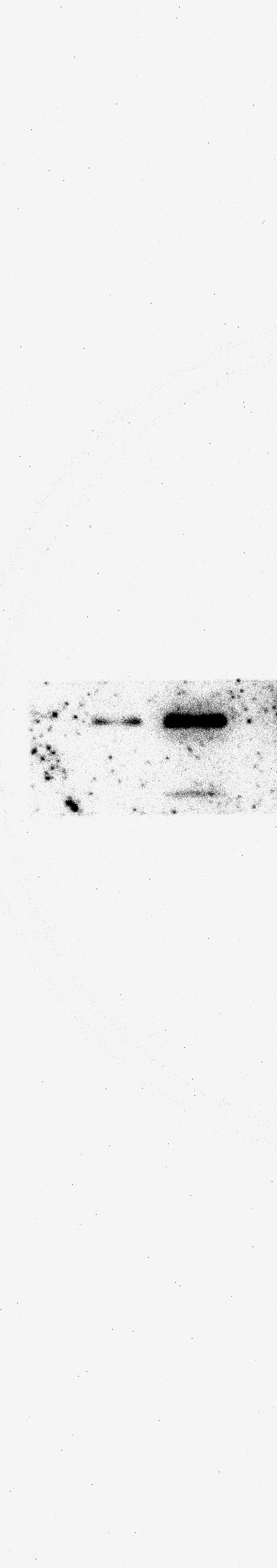

Supplement: Supplemental Information 1 [file peerj-11-14645-s001.zip › additional files/Figure 1E COMM10 (3).jpg]

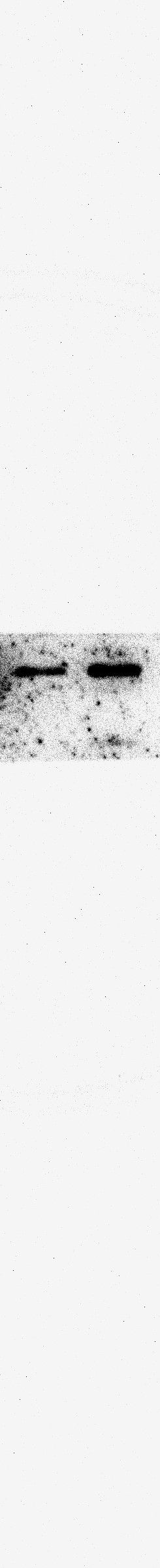

Supplement: Supplemental Information 1 [file peerj-11-14645-s001.zip › additional files/Figure 1E COMM10 (4).jpg]

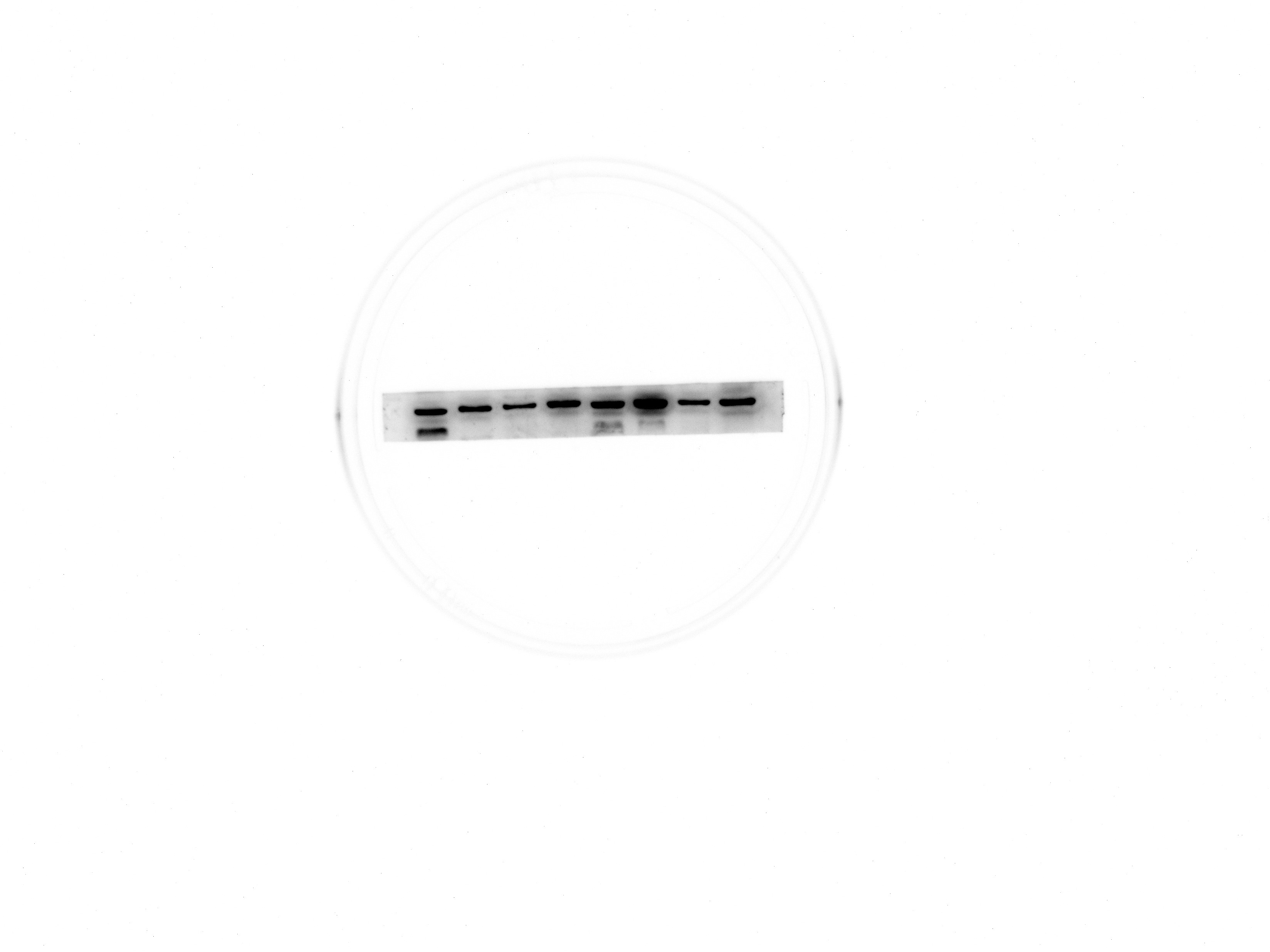

Supplement: Supplemental Information 1 [file peerj-11-14645-s001.zip › additional files/Figure 1E GAPDH (1).jpg]

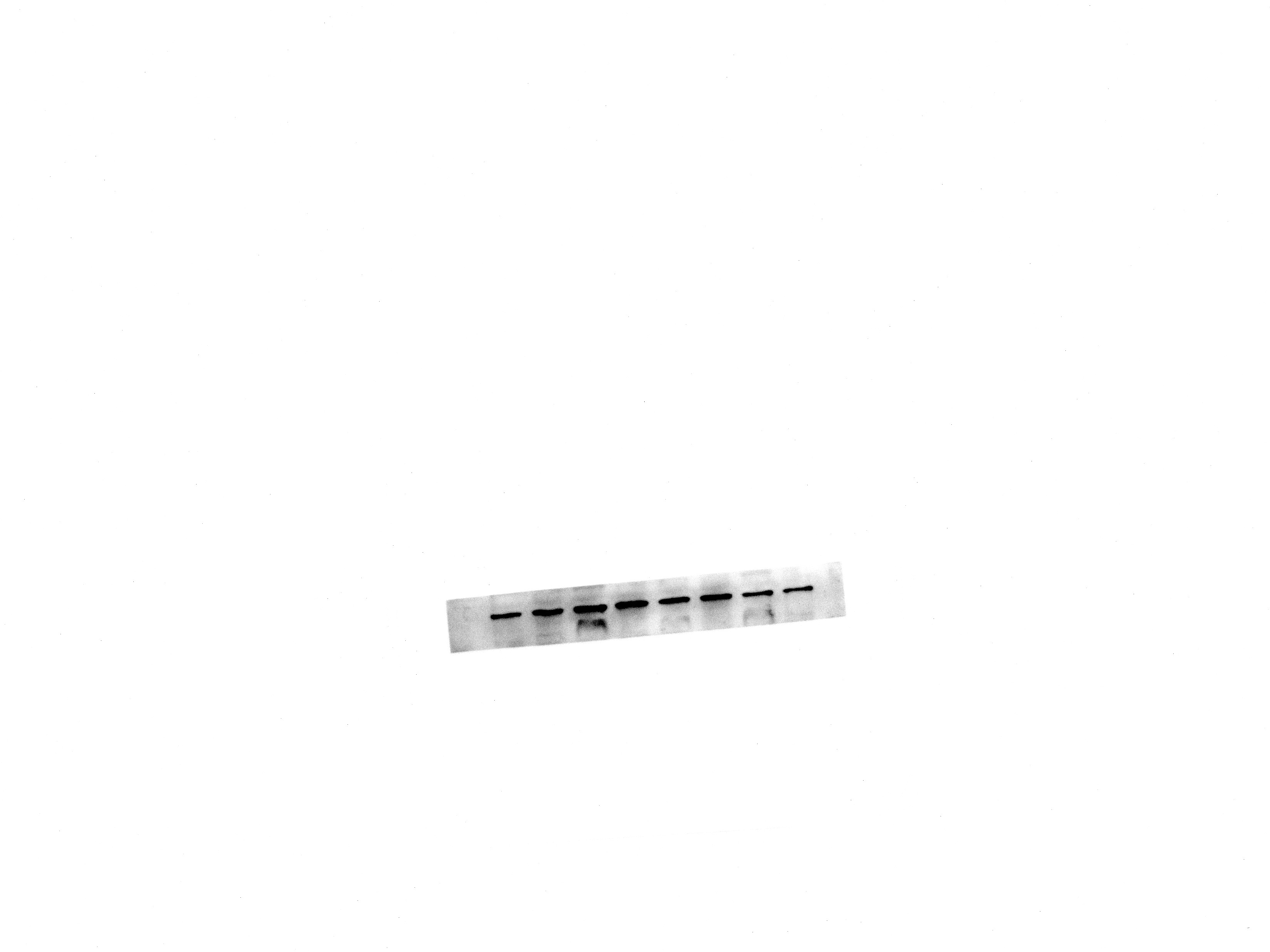

Supplement: Supplemental Information 1 [file peerj-11-14645-s001.zip › additional files/Figure 1E GAPDH (2).jpg]

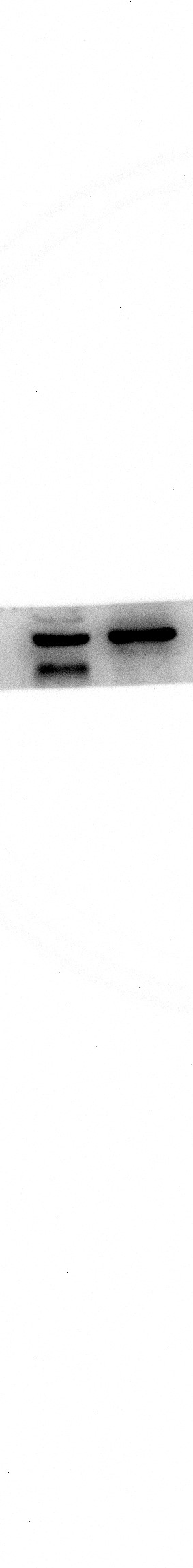

Supplement: Supplemental Information 1 [file peerj-11-14645-s001.zip › additional files/Figure 1E GAPDH (3).jpg]

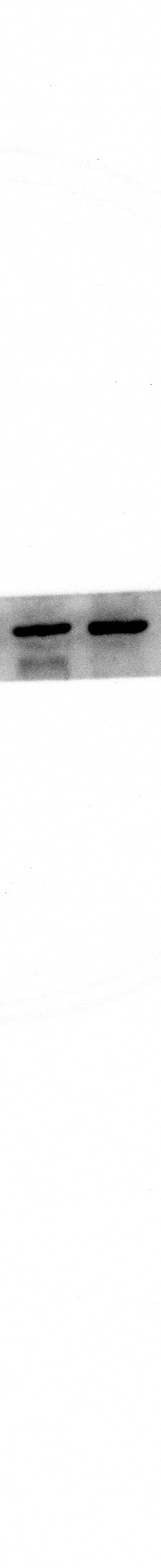

Supplement: Supplemental Information 1 [file peerj-11-14645-s001.zip › additional files/Figure 1E GAPDH (4).jpg]

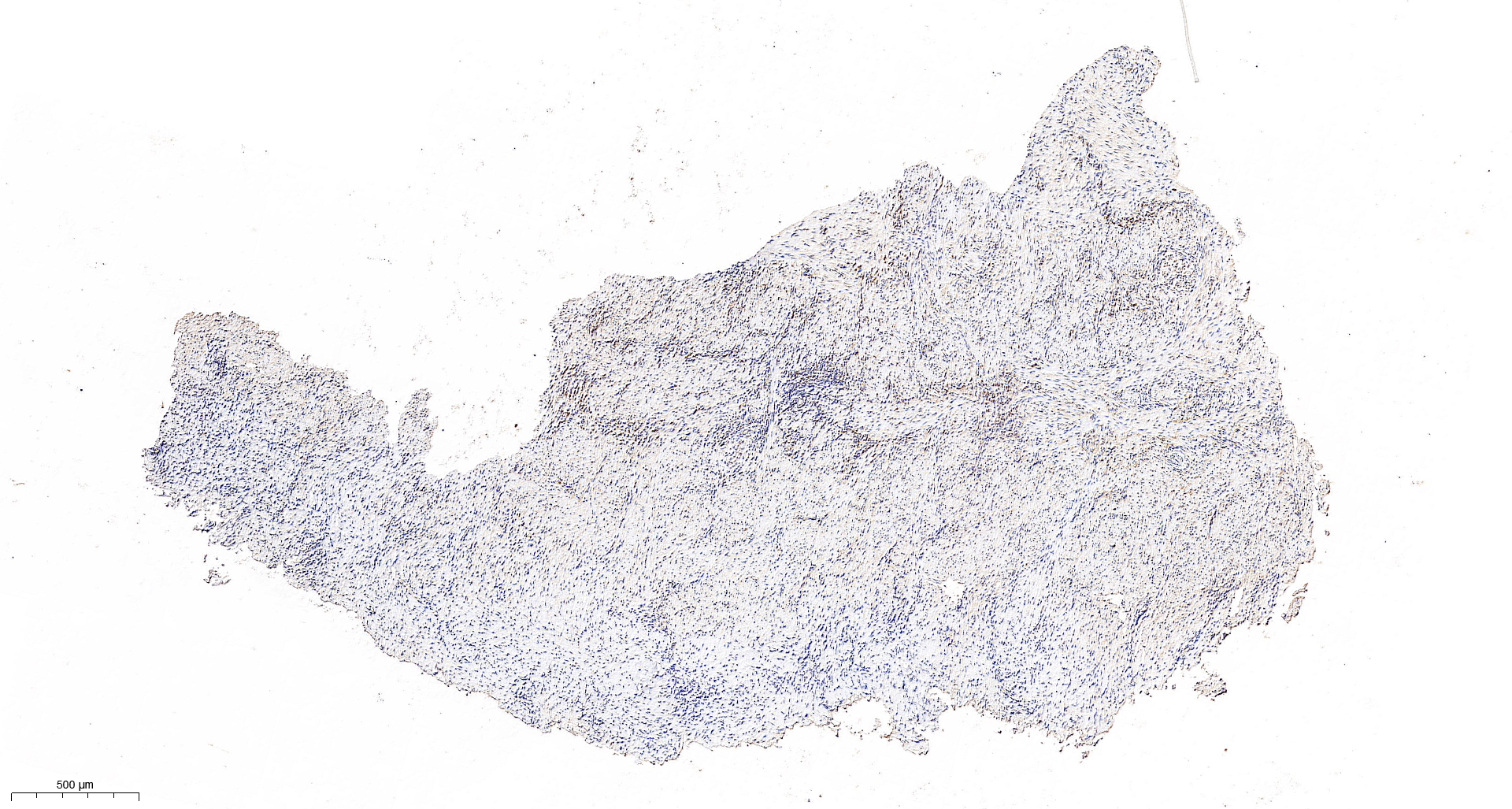

Supplement: Supplemental Information 1 [file peerj-11-14645-s001.zip › additional files/Figure 1F.jpg]

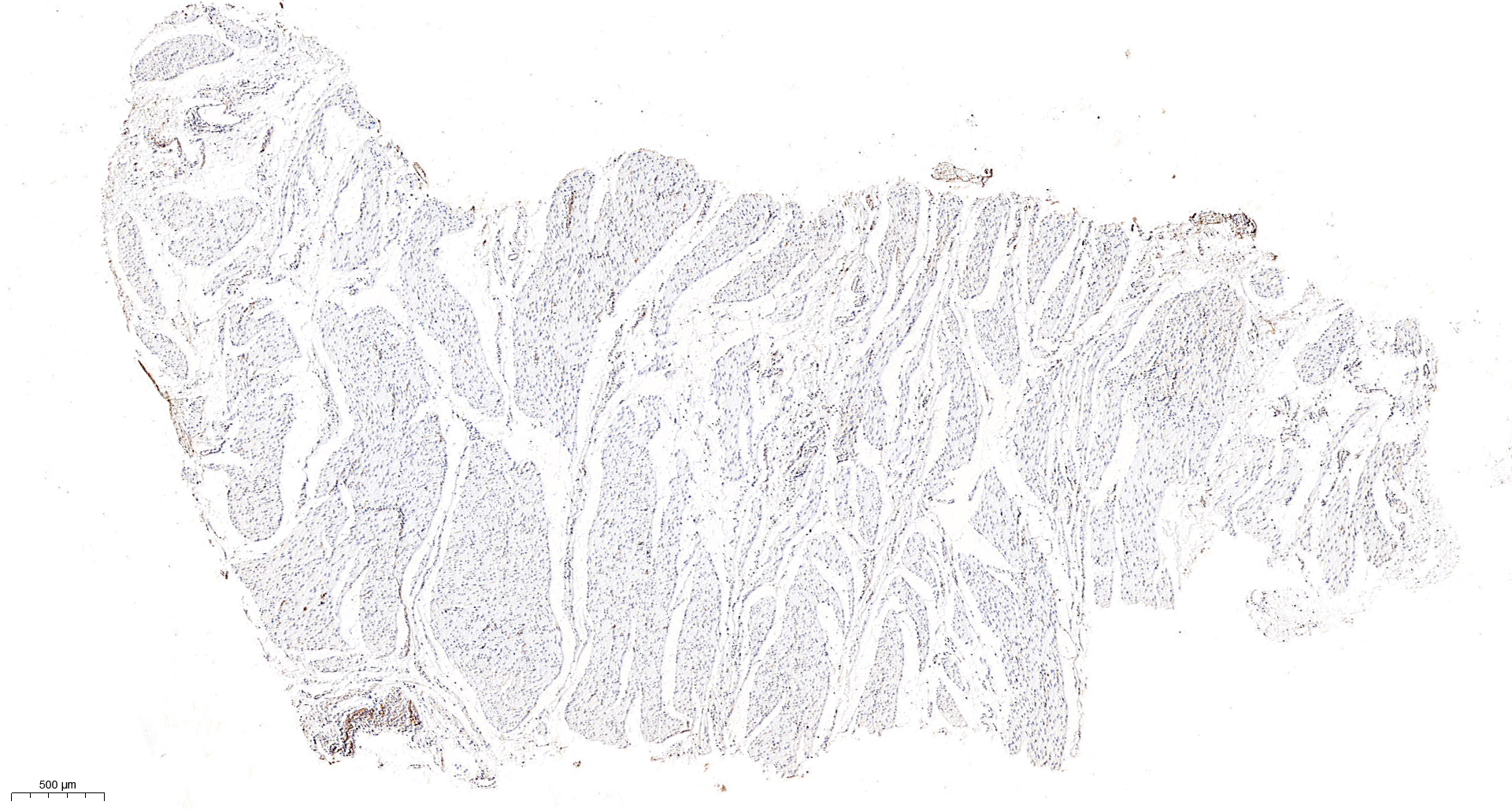

Supplement: Supplemental Information 1 [file peerj-11-14645-s001.zip › additional files/Figure 1G.jpg]

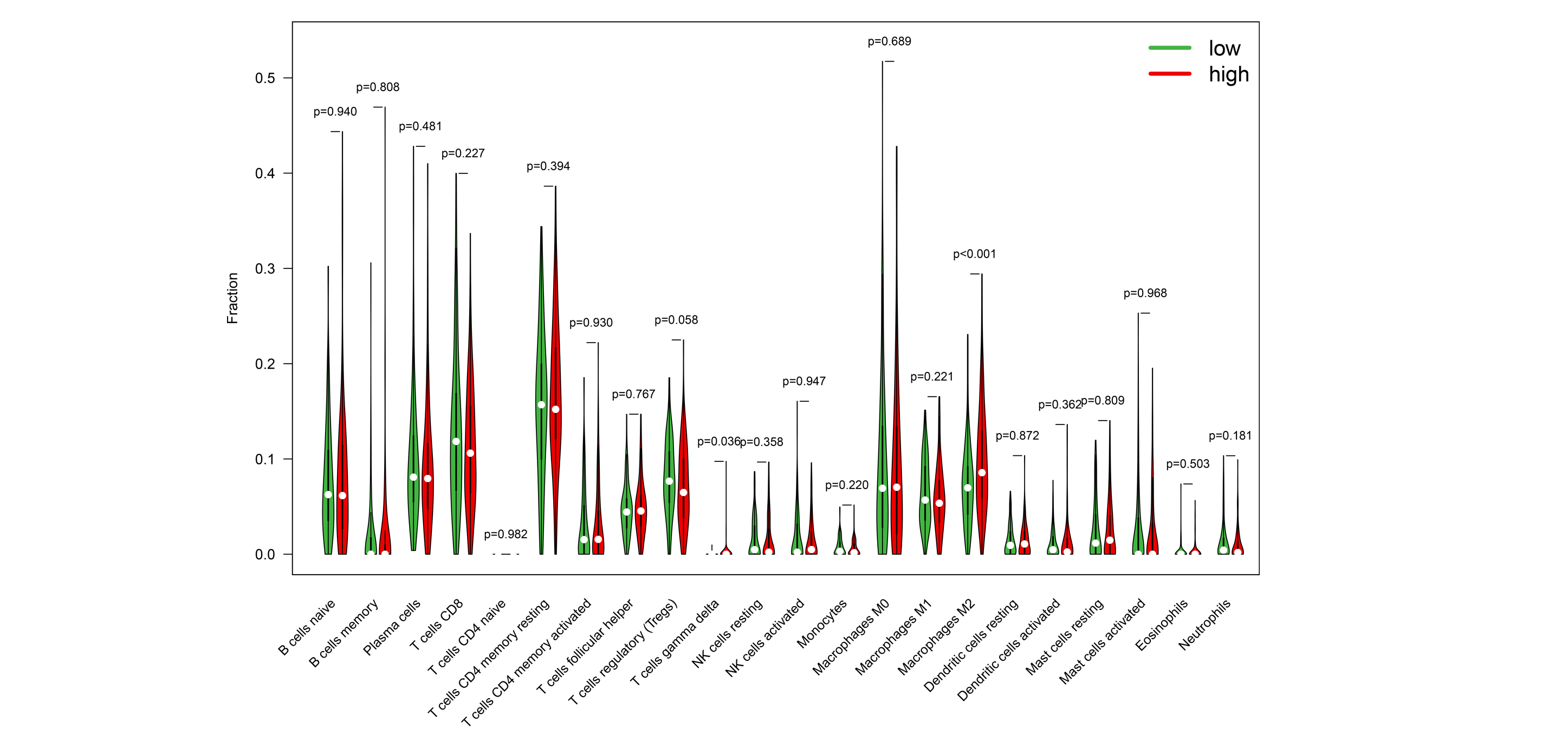

Supplement: Supplemental Information 2 [file peerj-11-14645-s002.png]
